# T90. MEMBERSHIP IN A SCHIZOTYPY TAXON PREDICTS HOPELESSNESS AND THOUGHTS OF SELF-HARM 7 YEARS LATER

**DOI:** 10.1093/schbul/sby016.366

**Published:** 2018-04-01

**Authors:** Richard Linscott

**Affiliations:** University of Otago

## Abstract

**Background:**

Expressions of liability for schizophrenia are associated with suicidal thinking and behaviour. This relationship appears not to be specific to different expressions of suicide, although there is evidence suggesting that schizophrenia liability is associated with greater lethality. The relationship is evident among help-seeking and non-help-seeking volunteers and using measures of psychosis experience or self-reported schizotypy; it persists despite controlling for other psychopathologies. Given these observations, the link between suicide and schizophrenia liability may be rooted in shared pathogenic mechanisms. With this in mind, I tested whether stress sensitivity could contribute to the link between schizophrenia liability and suicidality in a prospective study.

**Methods:**

At baseline (T1), n = 1074 undergraduates (M = 19.8 years, SD = 3.1; 30% male) completed the Schizotypal Personality Questionnaire (SPQ) and the Acute Hassles Scale (AHS), a self-report measure of stress sensitivity. Participants were classified as schizotypal or non-schizotypal by taxometric analyses of items for specific SPQ facets (cognitive-perceptual, interpersonal, and disorganization) and a general SPQ item set. Participants were classified as schizotypal (n = 43) if they were classified to the general schizotypy class to two or more specific-facet classes. At follow-up (T2) 7.8 years later (SD = 1.9, range = 5.5 to 10.5 years), the T1 schizotypy group (n = 43) and an age- and sex-matched control group (n = 216) were invited to participate in an online follow-up study. Of those invited, n = 84 (M = 27.7 years, SD = 3.3; 26% male; n = 15 schizotypal at T1) provided consent and completed the SPQ and AHS. At T2, hopelessness was assessed using 3 items from the Depression, Anxiety, and Stress Scales and thoughts of self-harm were assessed with one item from DSM-5 Self-Rated Level 1 Cross-Cutting Symptom Measure for adults. The small n at T2 prevented taxometric analyses of SPQ ratings at T2.

**Results:**

At T2, 9.5% of participants reported thoughts of self-harm and 17.8% hopelessness. Cross-sectional regression analyses of T2 data showed that the SPQ and AHS total scores each predicted concurrent self-harm thoughts (β = .52, p < .001, and β = .45, p < .001, respectively) and hopelessness (β = .43, p < .001, and β = .24, p < .05, respectively). When T2 SPQ and AHS were entered simultaneously, only SPQ scores predicted self-harm thoughts (β = .39, p = .001 for SPQ; β = .20, p = .10, for AHS) and hopelessness (β = .46, p = .001, for SPQ; β = -.04, p = .74 for AHS). In longitudinal analyses, T1 taxon membership predicted T2 self-harm thoughts (β = .28, p = .011) and hopelessness (β = .33, p = .002) but T1 AHS did not (β = .09, p = .43, and β = .09, p = .40, respectively). T1 taxon membership remained a significant predictor of T2 self-harm thoughts (β = .28, p = .016) and hopelessness (β = .34, p = .004) when T1 AHS was entered as a concurrent predictor, whereas AHS predicted neither outcome (β = -.01, p = .95, and β = -.02, p = .84, respectively).

**Discussion:**

Schizotypy classification during the late teens or early 20s predicted hopelessness and thoughts of self-harm 5 to 10 years later. Although stress sensitivity was correlated with concurrent thoughts or self-harm, stress sensitivity could not have accounted for the link between schizotypy and self-harm thoughts or hopelessness. The study is limited by the rudimentary nature of the assessment of self-harm thinking, the modest sample size, and the large rate of loss to follow-up.

